# Better Antiretroviral Therapy Outcomes at Primary Healthcare Facilities: An Evaluation of Three Tiers of ART Services in Four South African Provinces

**DOI:** 10.1371/journal.pone.0012888

**Published:** 2010-09-21

**Authors:** Geoffrey Fatti, Ashraf Grimwood, Peter Bock

**Affiliations:** 1 Kheth'Impilo, Cape Town, South Africa; 2 Lung Clinical Research Unit, University of Cape Town Lung Institute, Cape Town, South Africa; 3 Primary Healthcare Directorate, University of Cape Town, Cape Town, South Africa; Instituto de Medicina Tropical Alexander Von Humboldt, Peru

## Abstract

**Background:**

There are conflicting reports of antiretroviral therapy (ART) effectiveness comparisons between primary healthcare (PHC) facilities and hospitals in low-income settings. This comparison has not been evaluated on a broad scale in South Africa.

**Methodology/Principal Findings:**

A retrospective cohort study was conducted including ART-naïve adults from 59 facilities in four provinces in South Africa, enrolled between 2004 and 2007. Kaplan-Meier estimates, competing-risks Cox regression, generalised estimating equation population-averaged models and logistic regression were used to compare death, loss to follow-up (LTFU) and virological suppression (VS) between PHC, district and regional hospitals. 29 203 adults from 47 PHC facilities, nine district hospitals and three regional hospitals were included. Patients at PHC facilities had more advanced WHO stage disease when starting ART. Retention in care was 80.1% (95% CI: 79.3%–80.8%), 71.5% (95% CI: 69.1%–73.8%) and 68.7% (95% CI: 67.0%–69.7%) at PHC, district and regional hospitals respectively, after 24 months of treatment (*P*<0.0001). In adjusted regression analyses, LTFU was independently increased at regional hospitals (aHR 2.19; 95% CI: 1.94−2.47) and mortality was independently elevated at district hospitals (aHR 1.60; 95% CI: 1.30−1.99) compared to PHC facilities after 12 months of ART. District and regional hospital patients had independently reduced probabilities of VS, aOR 0.76 (95% CI: 0.59−0.97) and 0.64 (95% CI: 0.56−0.75) respectively compared to PHC facilities over 24 months of treatment.

**Conclusions/Significance:**

ART outcomes were superior at PHC facilities, despite PHC patients having more advanced clinical stage disease when starting ART, suggesting that ART can be adequately provided at this level and supporting the South African government's call for rapid up-scaling of ART at the primary level of care. Further prospective research is required to determine the degree to which outcome differences are attributable to either facility level characteristics or patient co-morbidity at hospital level.

## Introduction

In 2007 UNAIDS estimated that there were 4.9–6.6 million adults and children living with HIV/AIDS and that 270 000–420 000 people died annually from the disease in South Africa [1]. Although the South African Department of Health (DOH) has the largest antiretroviral treatment (ART) programme internationally [2], the 568 000 patients on treatment in mid-2008 represented only 40.2% of treatment-eligible adults at that time [3]. The South African National Strategic Plan for HIV/AIDS, published in 2007, targeted expanding ART coverage to ‘80% of those who need it by 2011’ [4]. The government's HIV/AIDS budgetary allocation for the corresponding period was however well below that required to reach this target [5], [6], and at the current rate of ART scale up an estimated 2.16 million people will die from the disease between 2007–2011 in South Africa [7].

The government implemented rollout of ART, initiated in 2004, has endorsed the World Health Organization's (WHO) simplified approach with the provision of standardised first and second line regimens provided along standardised clinical practice guidelines. The effectiveness of ART in reducing HIV/AIDS related morbidity and mortality globally and the need for rapid scale-up of ART services in South Africa is well described, and outcome data from individual sites and provinces compares well with results from other sub-Saharan countries and the developed world [8]–[11].

There is however a paucity of reliable government statistics and publications describing clinical outcomes of ART patients managed at routine primary healthcare (PHC) facilities outside the Western Cape province and there are no publications directly comparing adult programmatic outcomes between levels of care on a broad scale in South Africa. There have been conflicting reports of the effectiveness of ART delivery at PHC facilities when compared to hospital-based care in sub-Saharan Africa: Outcomes at PHC facilities were superior within a single sub district in Lusikisiki, South Africa [12]; but a recent Malawian study reported higher mortality rates amongst ART patients attending PHC facilities [13].

The aim of this study was to compare baseline characteristics and treatment outcomes between patients managed at different levels in the health system in a large routine patient cohort from four provinces in South Africa, representing over 7% of adults enrolled in public sector ART facilities between 2004 and 2007 in the country [14]. Additional objectives were to compare baseline characteristics and ART outcomes between patients in different provinces and to quantify baseline determinants of poor outcome after starting ART.

## Methods

### Ethics Statement

The study was approved by the University of Cape Town Health Sciences research ethics committee. Patient informed consent was not required as the data collected was routine operational monitoring data.

### Study design, setting and participants

A retrospective cohort study of adults enrolling for ART between 01 December 2004 and 31 December 2007 at 59 public health facilities in both urban and rural areas, including 47 PHC facilities, nine district hospitals and three regional hospitals was conducted. All sites were part of the government-run ART program and were supported by the nongovernmental organisation (NGO) Absolute Return for Kids (currently named Kheth'Impilo). Facility visits were free of charge for HIV positive patients. All sites supported by the NGO that had an electronic patient monitoring system were included in the study.

Twenty-eight sites were in the Western Cape province, nine in the Eastern Cape, 15 in KwaZulu-Natal and seven in Mpumalanga. There were two, three, three, and one district hospitals included in the above provinces respectively with an additional three regional hospitals in KwaZulu-Natal. The majority of patients lived in peri-urban informal settlements, but the Eastern Cape, KwaZulu-Natal and Mpumalanga also included patients from rural areas. Unemployment rates (2007) in the Western Cape, Eastern Cape, KwaZulu-Natal and Mpumalanga were 15.7%, 26.1%, 30.0% and 22.0% respectively, and crude death rates for these provinces were 9.8, 13.0, 11.5 and 11.8 deaths per 1000 population (2001) respectively [15]. The vast majority of patients accessing care at all levels of the healthcare system are impoverished, with lack of adequate sanitation and malnutrition being common. Sites started recruiting patients at various time-points between December 2004 and December 2007.

Adults with a CD4 cell count <200 cells/µL and/or a WHO stage IV defining illness were eligible to start treatment as per the South African national treatment guidelines. All ART-naïve adults (≥16 years) with documented date of birth, gender, date of starting ART and who initiated triple combination ART were eligible to be included in analyses.

Standardised first-line regimens consisted of two nucleoside reverse transcriptase inhibitors and one non-nucleoside reverse transcriptase inhibitor. Workup and maintenance of patients on ART was implemented according to the National Department of Health guidelines, which are standardized across facilities and between health system levels. Prior to starting ART, patients underwent clinical and immunological staging, and received HIV education and treatment adherence counselling. Patients on treatment had to attend monthly for clinical checks and to collect their treatment from pharmacies. CD4 cell count was measured at ART initiation and at six monthly intervals, and viral load was monitored six-monthly on treatment. Laboratory monitoring was performed by the South African National Health Laboratory Services, which were off-site except in the case of larger hospitals.

Patients were followed-up until 31 March 2008 or until the NGO exited from a site (18 sites in the Western Cape, one in KwaZulu-Natal and one site in the Eastern Cape were exited between March 2005 and February 2008). Outcome measures were death, loss to follow-up (LTFU) and virological suppression (VS). Deaths were reported by the clinic staff or the patient's community-based adherence counsellor. LTFU was defined as no visit to the clinic for three months or more beyond the last missed appointment date and the patient was not known to have died or moved to another treatment program or place. Patients who were late for appointments or LTFU were attempted to be traced at home by adherence counsellors. A conservative measurement of time was used, being from the date of ART initiation until the date of last follow-up visit. In time-to-event statistical analyses, follow-up times of patients who transferred to other facilities were censored at the date of last visit.

### Data collection and statistical analyses

Individual-level patient data was collected prospectively for routine monitoring purposes by designated site-based data capturers using Microsoft Access databases, which was pooled on a quarterly basis to a central data warehouse using standard operating procedures. Continual data cleaning and quality control routines were implemented to enhance data validity.

Groups were compared using the Kruskal-Wallis and Pearson's χ2 tests for continuous and categorical data respectively. Virological suppression was calculated as the proportion of patients with a viral load <400 copies/ml with binomial exact confidence intervals. Kaplan-Meier estimates of mortality and retention in care (RIC) were calculated. An intention-to-treat analysis was performed ignoring treatment stoppages or interruptions. The logrank test was used to compare groups. The temporal distribution of deaths and LTFU were assessed by plotting smoothed instantaneous hazard curves from product-limit analyses, using weighted kernel density estimates based on an Epanechnikov function.

Multivariable competing-risks Cox proportional hazards regression models were used to examine baseline factors associated with time to death and LTFU after 12 months of ART, including only patients with available baseline CD4 cell count and WHO clinical staging. Competing-risks regression accounts for the fact that the risks of death and LTFU may not be independent, and allows the baseline hazard to differ between the competing risks [16]. Regression models were adjusted for all available baseline characteristics, controlled for heterogeneity due to different site cohorts, stratified by event type, and robust variance adjustment was used due to the twin strata for death and LTFU. Proportional hazards assumptions were confirmed not to be violated using scaled Schoenfeld residuals and log-log plots of survival against analysis time for each of the covariates.

Multivariable generalised estimating equation population-averaged models were used to analyse the effect of patient and available site-level factors associated with VS up to 24 months of ART, adjusting for available baseline variables and time on ART. An exchangeable working correlation structure was selected and the results verified to be similar assuming other applicable correlation structures. A sensitivity analysis using multivariable logistic regression of baseline factors associated with VS at 12 months was performed, to assess if results were consistent. All analyses were performed with Stata version 9.2 (College Station, Texas, USA).

## Results

### Baseline characteristics

A total of 42 237 patient database records were screened for eligibility for the study and 29 203 patients were included in the analysis. Patients were excluded from the analysis for the following reasons: 3581 had missing demographic data, 3733 were ART-experienced, 2147 were aged <16 years when starting ART and 3573 started ART after 31 December 2007.

The median age was 34.3 years (IQR: 29.4–40.7) and 68.1% were women (table 1). The median baseline CD4 cell count was 114 cells/µL (IQR: 57–166) and increased marginally from 111 cells/µL (IQR 54–170) in 2004 to 118 cells/µL (IQR 61–170) in 2007. Overall, 75.8% of patients had a baseline WHO clinical stage of III or IV.

**Table 1:** Baseline characteristics of adults starting ART stratified by facility levels.

	All	Primary healthcare	District Hospitals	Regional Hospitals	*P-*value
**Number of patients, n (%)**	29 203 (100)	19 273 (66.0)	2483 (8.5)	7447 (25.5)	
**Number of sites**	59	47	9	3	
**Mean patients enrolled/site, n**	495	410	311	2482	
**Median age, y (IQR)**	34.3 (29.4–40.7)	34.0 (29.2–40.4)	34.7(29.9–41.6)	34.8 (29.7–41.5)	0.001
**Male, n (%)**	9 317 (31.9%)	6164 (32.0)	779 (31.4)	2374 (31.9)	0.83
**WHO stage ≥III, n (%)** a	16 714 (75.8)	11 515 (79.3)	934 (58.3)	4265 (72.2)	<0.001
**Unstaged, n (%)** b	7 163 (24.5)	4774 (24.6)	882 (35.5)	1537 (20.6)	<0.001
**Baseline CD4 cell count (cells/µL), median (IQR)**	114 (I57–166)	113 (57–165)	109 (54–155)	116 (57–170)	0.001
**CD4 cell count unavailable, n (%)** b	5734 (19.6)	4367 (22.7)	517 (20.8)	850 (11.4)	<0.001
**Western Cape patients, n (%)** c	11 557 (39.6)^b^	10 708 (92.7)	849 (7.3)	0 (0)	
**Eastern Cape patients, n (%)** c	1583 (5.4)^b^	1255 (79.3)	328 (20.7)	0 (0)	
**KwaZulu-Natal patients, n (%)** c	15 540 (53.2)^b^	6823 (43.9)	1270 (8.2)	7447 (47.9)	
**Mpumalanga patients, n (%)** c	523 (1.8)^b^	487 (93.1)	36 (6.9)	0 (0)	

a Percentage of available results.

b Percentage of total patients.

c Percentage of patients per province.

IQR-interquartile range; WHO-world health organization.

The number of patients enrolled at PHC facilities, district hospitals and regional hospitals were 19 273 (66%), 2483 (8.5%) and 7447 (25.5%) respectively. The mean number of patients enrolled per facility at regional hospitals was 2482 compared to 410 at PHC sites. Baseline CD4 cell counts were similar between facility levels, however patients enrolling at PHC sites had more severe clinical stage disease (*P*<0.001), table 1.

The number of patients enrolled in the Western Cape, Eastern Cape, KwaZulu-Natal and Mpumalanga provinces were 11557 (39.6%), 1583 (5.4%), 15 540 (53.2%) and 523 (1.8%) respectively with corresponding median baseline CD4 cell counts of 112 cells/µL (IQR: 54–165), 128 cells/µL (IQR: 69–180), 113 cells/µL (IQR: 57–164) and 125 cells/µL (IQR: 59–199). The Western and Eastern Cape had the highest proportions with advanced clinical disease, with 79.0%, 79.0%, 73.8% and 74.4% of patients having a baseline WHO stage of III or IV in the Western Cape, Eastern Cape, KwaZulu-Natal and Mpumalanga respectively (*P*<0.001).

**Figure 1:**
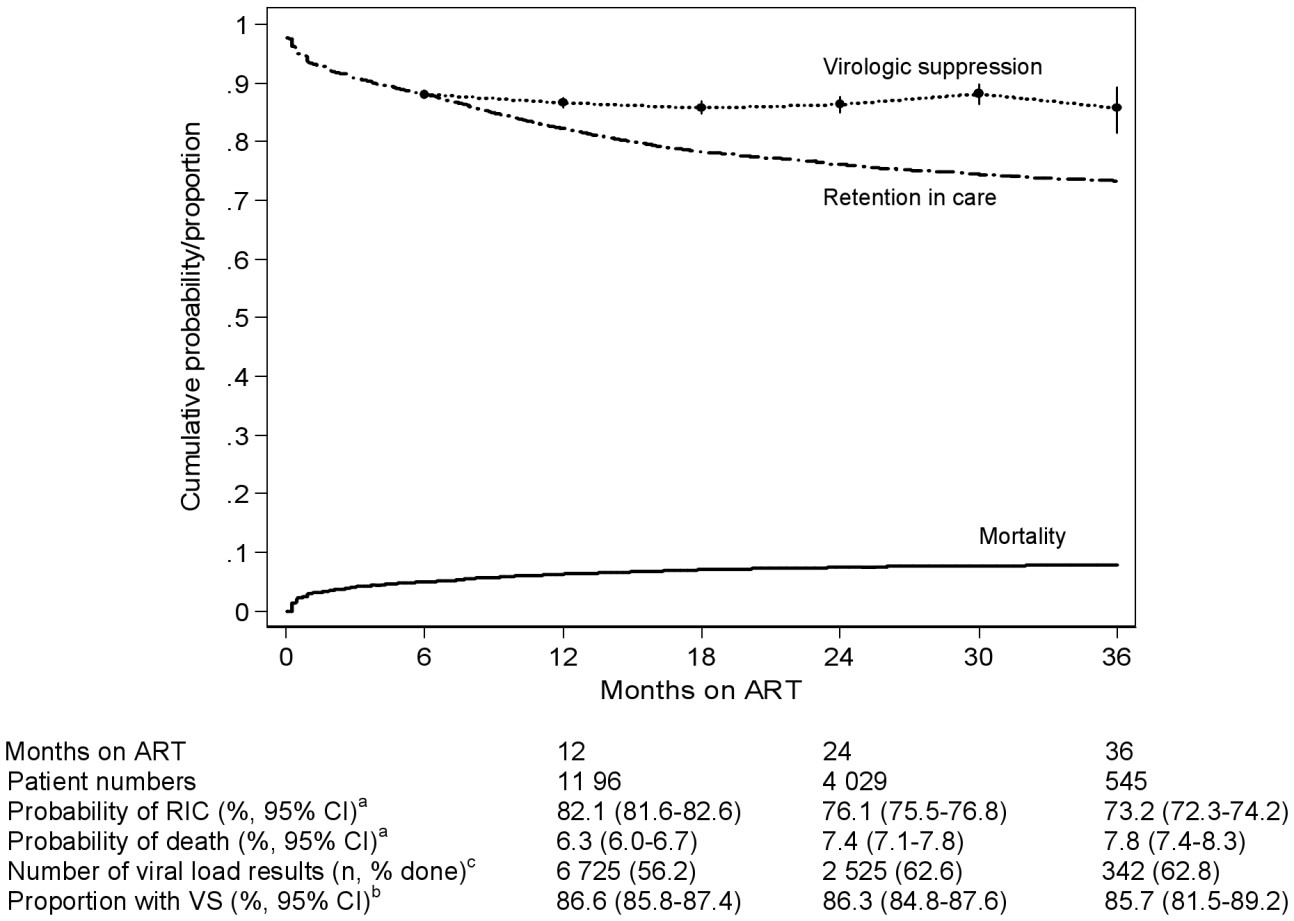
Kaplan-Meier estimates of death, retention-in-care; and proportions virologically suppressed adults according to duration of ART. ^a^ Cumulative probabilities with Greenwood point-wise 95% confidence intervals. ^b^ Proportions with binomial exact 95% confidence intervals. ^c^ Proportion of patients eligible for viral load tests with a recorded database result. RIC, Remaining in care; VS, Virologic suppression (<400 copies/ml).

**Figure 2:**
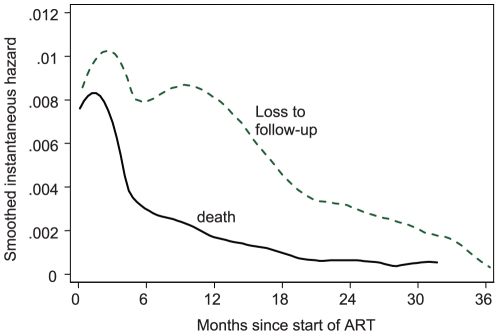
Smoothed hazard estimates of death and loss to follow-up according to duration of ART.

Over 29 297 person-years of observation 1656 (5.7%) patients died after starting ART, 3401 (11.7%) were LTFU and 1606 (5.5%) transferred-out to other facilities. Following 24 months of treatment, the overall cumulative probability of reported mortality was 7.4% (95% CI: 7.1%–7.8%) and patient RIC was 76.1% (95% CI: 75.5%–76.8%), figure 1. The proportion of patients LTFU after 24 months was 12.0% (95% CI: 11.1%–12.8%). The hazard of death decreased dramatically after the first six months of treatment (figure 2), with mortality rates being 11.1 (95% CI: 10.5–11.7) and 2.1 (95% CI: 2.0–2.2) deaths/100 person-years during months 0–6 and 6–24 of treatment respectively. The hazard of LTFU was also highest initially, but decreased progressively as the duration of treatment increased.

The proportion of eligible patients having available viral load results in the database varied from 56.2% at 12 months to 62.8% at 36 months. Overall virological suppression was good; being greater than 85% at all six-monthly measurement intervals up to 36 months of treatment (figure 1). Compared to patients aged ≥20 years, VS in young adults aged 16–19 years was reduced, being 74.6% (95% CI: 62.5%–84.5%); *P* = 0.001) and 68.1% (95% CI: 52.8 –80.9%; *P*<0.001) at 6 and 12 months respectively. There were more women amongst this age group (89.1% versus 68.0% in ages ≥20 years; *P*<0.001) however there were no other differences in baseline clinical or demographic variables between these age categories.

### Comparison of ART outcomes between treatment facility levels and patient factors associated with poor outcomes

**Figure 3:**
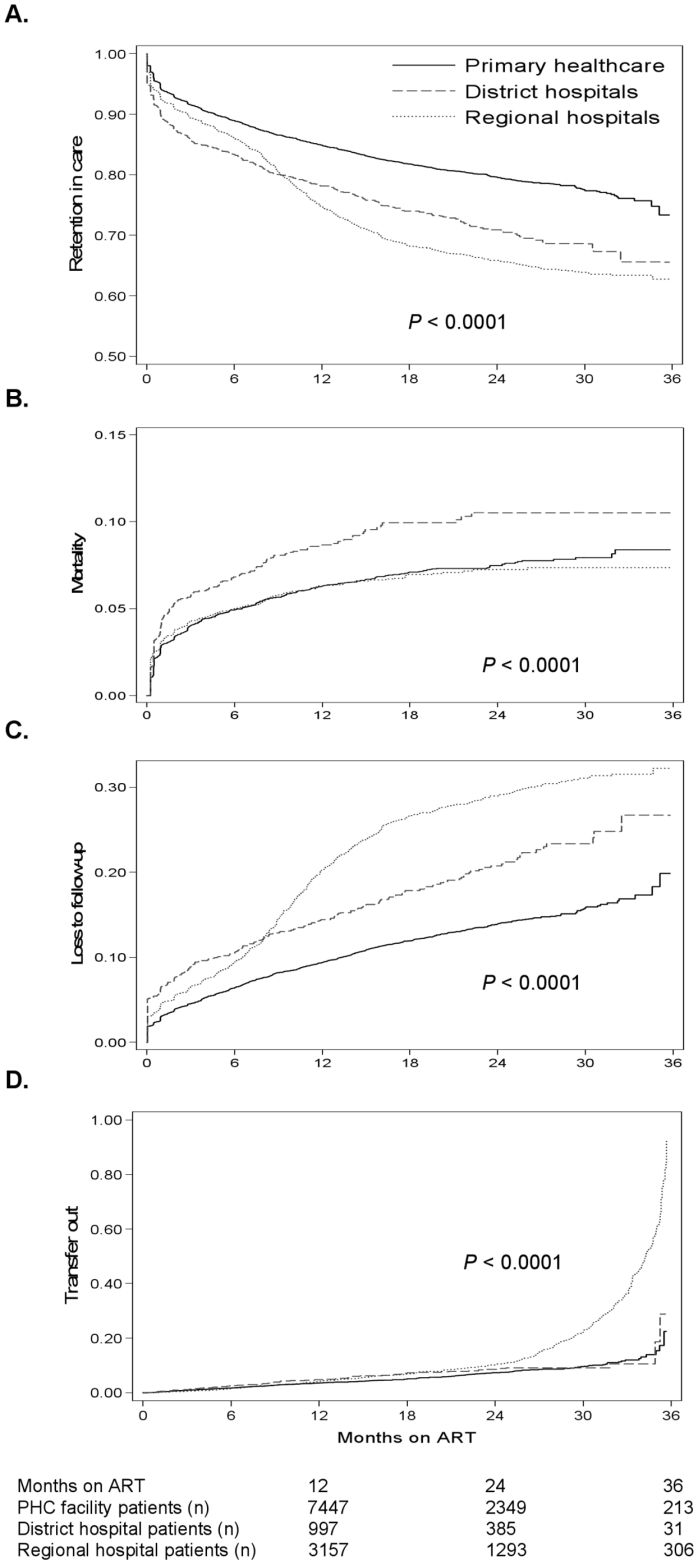
Cumulative probabilities of retention-in-care (A), mortality (B), loss to follow-up (C), and transfer-out (D) by facility level.

During the study period, 1076 (5.6%), 182 (7.3%) and 398 (5.3%) patients died (*P* = 0.001), and 1719 (8.9%), 348 (14.0%) and 1334 (17.9%) become LTFU (*P*<0.001) at PHC facilities, district and regional hospitals respectively. Figure 3 compares Kaplan-Meier cumulative estimates of RIC, mortality, LTFU and transfer-out between levels of care. RIC was superior at PHC facilities, being 80.1% (95% CI: 79.3%–80.8%) compared to 71.5% (95% CI: 69.1%–73.8%) at district and 68.7% (95% CI: 67.0%–69.7%) at regional hospitals after 24 months of ART (logrank *P*<0.0001). Mortality was highest at district hospitals, being 10.3% (95% CI: 8.9%–12.0%) compared to 7.3% (95% CI: 6.9%–7.8%) at PHC facilities and 6.8% (95% CI: 6.1%–7.5%) at regional hospitals after 24 months (logrank *P*<0.0001). LTFU was lowest at PHC sites and highest at regional hospitals (logrank *P*<0.0001). Transfer-out increased dramatically after 24 months at regional hospitals.

Table 2 shows multivariable regression estimates of baseline factors associated with death and LTFU after 12 months of treatment in patients with all baseline measurements available (*n* = 18 866). There were no significant differences in baseline demographic variables between the full cohort and this subgroup; however more patients were treated at regional hospitals in the subgroup, with 12 268 (65.0%), 1330 (7.1%) and 5268 (27.9%) patients treated at PHC, district and regional hospitals respectively.

**Table 2:** Multivariable competing-risks Cox proportional hazards model of factors associated with death and loss to follow-up after 12 months of ART (*n* = 18 866)^a^.

	Death			Loss to follow-up		
Patient Factor	Adjusted Hazard Ratio	95% CI	*P*-value	Adjusted Hazard Ratio	95% CI	*P*-value
Male gender	1.14	1.00–1.30	0.047	1.20	1.09–1.33	0.001
Age^b^	1.06	0.99–1.15	0.104	0.89	0.84–0.94	<0.001
WHO stage 1/II^c^	1.00			1.00		
stage III	1.85	1.47–2.32	<0.001	1.15	1.01–1.32	0.036
stage IV	4.84	3.72–6.31	<0.001	1.97	1.67–2.34	<0.001
**Baseline CD4 cell count (cells/µL):**						
>200^c^	1.00			1.00		
50–199	1.61	1.09–2.38	0.017	1.31	1.07–1.62	0.009
25–49	3.07	2.00–4.75	<0.001	1.51	1.18–1.92	0.001
<25	3.80	2.48–5.82	<0.001	1.49	1.17–1.89	0.001
**Year of starting ART:**						
2004^c^	1.00			1.00		
2005	0.87	0.67–1.14	0.332	3.05	2.09–4.44	<0.001
2006	0.69	0.53–0.90	0.006	6.78	4.59–9.72	<0.001
2007	0.49	0.37–0.65	<0.001	6.18	4.30–8.89	<0.001
**Province**						
Western Cape^c^	1.00			1.00		
Eastern Cape	1.77	1.28–2.44	0.001	0.90	0.67–1.19	0.454
KwaZulu-Natal	1.42	1.20–1.67	<0.001	0.92	0.80–1.06	0.243
Mpumalanga	2.70	1.37–5.33	0.004	1.56	0.89–2.74	0.121
**Facility level**						
Primary healthcare^c^	1.00			1.00		
District hospitals	1.60	1.30–1.99	<0.001	1.36	1.11–1.66	0.002
Regional hospitals	1.07	0.91–1.27	0.376	2.19	1.94–2.47	<0.001

a Models are adjusted for all variables shown.

b 10-year age increase.

c Reference category.

WHO-World Health Organisation; CI-confidence interval; ART-antiretroviral therapy.

Compared to PHC sites, mortality was independently elevated at district hospitals (aHR 1.60; 95% CI: 1.30–1.99). LTFU was independently elevated at regional hospitals, aHR 2.19 (95% CI: 1.94–2.47). Patients in the Eastern Cape, KwaZulu-Natal and Mpumalanga provinces had independently increased probabilities of mortality compared to the Western Cape. Adjusted probabilities of LTFU between provinces were similar. WHO stage IV disease was associated with an increased probability of death (aHR 4.84; 95% CI: 3.72–6.31) as was decreasing CD4 cell count, with the risk being greatest with CD4 cell counts <25 cells/µL (aHR 3.80; 95% CI: 2.48–5.82). Older age was inversely related to the risk of LTFU. Patients enrolling in later years had considerably elevated risks of LTFU compared to patients enrolled in 2004, aHR 6.18 (95% CI: 4.30–8.89; 2007 cohort).

The median CD4 cell increase after twelve months of treatment was 159 cells/µL (IQR: 81–254), with no difference between PHC sites and hospitals (*P* = 0.44). PHC sites had considerably greater proportions of patients having available viral load results, with 73.0% (*n* = 5657), 29.4% (*n* = 293) and 25.4% (*n* = 803) of viral load results being available at 12 months at PHC, district and hospital facilities respectively. Overall VS was highest at PHC facilities, being 88.0% (95% CI: 87.6%–88.4%, *n* = 22 320) compared to 86.9% (95% CI: 84.8%–88.8%, *n* = 1141) and 82.2% (95% CI: 81.0%–83.4%, *n* = 4615) at district and regional hospitals respectively at any time-point on treatment, *P*<0.001.

Overall VS was highest in the Western Cape being 89.5% (95% CI: 88.9%–90.1%, *n* = 10 443) compared to 83.7% (95% CI: 82.1%–85.2%, *n* = 2187), 86.3% (95% CI: 85.7%–86.8%, *n* = 14 264) and 78.9% (95% CI: 75.1%–82.3%, *n* = 535) in the Eastern Cape, KwaZulu-Natal and Mpumalanga respectively at any time-point on treatment, *P*<0.001.

**Figure 4:**
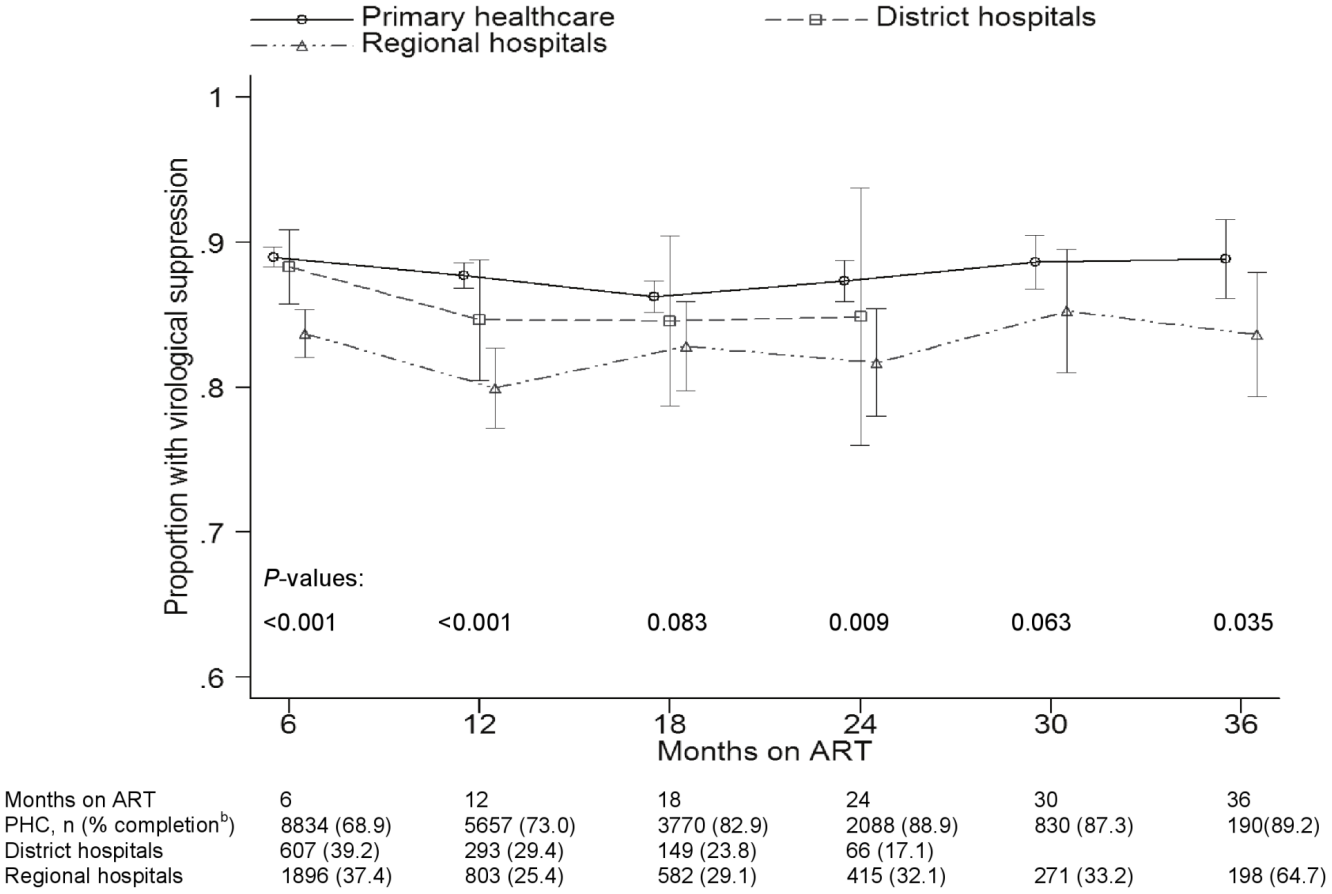
Proportions of patients with virological suppression at primary healthcare facilities and hospitals.^a^ ^a^ Vertical bars indicate 95% binomial exact confidence intervals. ^b^ Proportion of patients eligible for viral load tests with a recorded database result. PHC-primary healthcare facilities.

Figure 4 compares virological suppression between facility levels. A multivariable model predicting virological suppression at any follow-up duration up to 24 months of ART adjusting for baseline variables and duration of ART was derived, which included 10 458 patients having viral load data and all baseline values available. Of these, 7914 (75.7%), 445 (4.3%) and 2099 (20.1%) were from PHC facilities, district and regional hospitals respectively. District and regional hospital patients were found to have independently reduced probabilities of VS compared to PHC facility patients, aOR 0.76 (95% CI: 0.59−0.97) and 0.64 (95% CI: 0.56–0.75) respectively (table 3). In addition, young adults aged 16–19 years had a reduced probability of VS compared to those aged 20–29 years (aOR 0.44; 95% CI: 0.27–0.72). Patients aged ≥40 years had a higher probability of VS (aOR 1.69; 95% CI: 1.33–2.16). Patients in the Eastern Cape, KwaZulu-Natal and Mpumalanga provinces had independently reduced probabilities of VS compared to the Western Cape.

**Table 3:** Multivariable generalized estimating equation population-averaged model of virological suppression up to 24 months of ART (*n* = 10 458)^a^.

Variable	Adjusted OR	95% CI	*P*-value
Male gender	0.89	0.80–1.00	0.051
Age: 16–19 years	0.44	0.27–0.72	0.001
20–29 years^b^	1.00		
30–39 years	1.12	0.99–1.26	0.069
≥40 years	1.69	1.33–2.16	<0.001
WHO stage: I/II^b^	1.00		
III/IV	0.86	0.76–0.97	0.018
Baseline CD4 cell count (µL)	1.00	0.99–1.00	0.149
Western Cape province^b^	1.00		
Eastern Cape province	0.41	0.32–0.52	<0.001
KwaZulu-Natal province	0.69	0.59–0.82	<0.001
Mpumalanga province	0.30	0.17–0.51	<0.001
Primary healthcare facilities^b^	1.00		
District hospitals	0.76	0.59–0.97	0.030
Regional hospitals	0.64	0.56–0.75	<0.001
ART duration 6 months^b^	1.00		
ART duration 12 months	0.83	0.78–0.92	<0.001
ART duration 18–24 months	0.83	0.73–0.95	0.007

a Estimates adjusted for all variables shown and year of starting ART.

b Reference category.

WHO-World Health Organisation; CI-confidence interval; ART-antiretroviral therapy.

In sensitivity analyses of baseline factors associated with VS at 12 months adjusting for all available baseline measurements (*n* = 4885), district and regional hospitals patients similarly had reduced probabilities of VS compared to PHC facility patients, aOR 0.73 (95% CI: 0.49−1.09) and 0.58 (95% CI: 0.45–0.75) respectively. Young adults aged 16–19 years also had independently reduced probability of VS compared to those aged 20–29 years, aOR 0.26 (95% CI: 0.12–0.55). Patients outside the Western Cape similarly had independently reduced probabilities of VS; aOR 0.41 (95% CI: 0.27–0.60), aOR 0.72 (95% CI: 0.56–0.94) and aOR 0.20 (95% CI: 0.09–0.41) for the Eastern Cape, KwaZulu-Natal and Mpumalanga respectively compared to the Western Cape.

## Discussion

This is one of the first large-scale direct comparisons of the effectiveness of routine adult ART provision between health system levels at facilities across multiple provinces in South Africa. Overall outcomes were found to be superior at PHC clinics when compared to hospitals, despite PHC patients having more advanced clinical stage disease when starting ART. These findings concur with a South African study that compared outcomes between children initiated on ART at PHC clinics and hospitals within the Western Cape province [17].

Overall programmatic outcomes are comparable to published data from research sentinel sites in South Africa as well as with developed world cohorts [9]–[11] and the results support the previously observed trend toward increased LTFU amongst patients enrolled in more recent years in low-income settings [8], [18]. The most important reason for this trend is likely to be the increase in patient numbers at individual sites over time, with some clinics managing over 3000 ART patients; however it may also reflect that patients LTFU in earlier years had a greater probability of re-entering treatment than patients LTFU nearer the end of the study period. The steady increase in LTFU is concerning in the context of massive scale-up over the next 5–10 years and the paucity of published data detailing outcomes amongst patients in developing countries after five years of treatment.

Although facility-level quality of care variables were not directly measured in this study, the proportions of patients with available on-treatment viral load measurements was higher at PHC sites, and this may be used as a proxy indicator for site-based quality of care [8]. Facility-level factors that may negatively impact patient outcomes at hospital-based outpatient services include higher patient loads and high patient/staff ratios, which lead to reduced individual patient attention from nurses and counsellors and a resultant decreased quality of care, increased LTFU and treatment failure. Environmental factors that may affect outcomes between levels of care include the distance, difficulty and cost of transport to health facilities [19], [20]. Access to care at the primary level is likely to be easier and cheaper for patients, with services being more aligned to ongoing patients' needs and a probable decreased time to treatment initiation.

The independently higher risks of mortality and treatment failure amongst patients in the Eastern Cape, KwaZulu-Natal and Mpumalanga is a concern and is not explained by differences in baseline clinical or immunological parameters between these provinces. The reasons for this are probably multi-factorial and include differences in socioeconomic status, access to healthcare services as well as differences in the quality of health service delivery including patient/staff ratios. Further research is needed to explain and validate these results including larger sample sizes from the Eastern Cape and Mpumalanga.

Poor virological response is an important factor associated with disease progression and death, predisposes to the development of drug-resistance and reduces future treatment options [21]. Although overall virologic suppression was high and compared favourably with other studies [9], [10], young adults in this data are shown to have a greater risk of virologic failure. This trend is in keeping with other cohorts from South Africa and Haiti [22], [23], which demonstrated an association with poor adherence, highlighting the need for specific interventions to improve adherence in this age group.

The failure of the median baseline CD4 cell count in this dataset to increase substantially over time is concerning and contrasts with other published data from South Africa covering the same time period [24]. This may reflect that as the ART program expanded into new areas over time, the baseline CD4 cell counts of new patients starting treatment in the previously unreached areas remained relatively low, and implies that there remains a large number of people who urgently require ART in the country. Given the recognition of temporal trends in baseline CD4 cell count as a proxy measure of access and uptake of treatment, the poor increase of this data element raises concerns of the impact of the national ART program on broader demographic outcomes in the country and supports the argument for urgent expansion and scale-up of ART services [8], [25].

The rapid expansion of quality ART programs within the limited budget envelope in South Africa will require the implementation of innovative and cost-effective approaches to service delivery. Several obstacles to the rapid scale-up of ART services have been identified at sites incorporated in this analysis, including underutilisation of PHC clinics, rapid increases in patient numbers leading to overcrowding of individual sites, as well as an over-reliance on doctor-driven services. Rapid scale-up necessitates broadening of services to the primary healthcare level facilitating both initiation of new patients and down-referral of stable patients from hospitals and large community health centres to smaller PHC clinics as has been effective in the Lusikisiki sub-district [12].

Nurse-monitored services have recently been shown to be effective in an observational study in Lesotho [26] and non-inferior to doctor-monitored care in a randomised non-inferiority trial [27]. Nurses are however also in short supply and under pressure [28], and expanding elements of service delivery to the community should be explored. Several programmes in South Africa have had success with the use of community workers to provide adherence support to ART patients, however the full utility of this cadre of health worker has not been maximally developed or evaluated. Simple health systems interventions, such as streamlining of patient flow within sites and increasing the time between clinic appointments or pharmacy pick-up dates for stable patients (in order to decrease the daily patient load thus improving service quality at each site) may also be effective in facilitating rapid scale-up of quality services and should be prioritised under the umbrella of clinical governance.

Maintaining good monitoring systems becomes more critical and more complex as the numbers of treatment providers, patients on treatment and duration of treatment increases. The paper-based system rolled out by the Western Cape DOH is the more referenced in South Africa [8], [17] due to the failure to successfully implement an electronic ART monitoring systems across South Africa. The national DOH is currently piloting a number of electronic systems to identify an appropriate electronic solution for monitoring the rollout nationally. Electronic systems have however produced poor quality data in a number of cohorts in low-income settings, and require sufficient resources and adequately trained data staff to ensure adequate data quality [29].

The strengths of this study are that pooled data from a large number of patients and sites in different settings were used, and individual-level data was collected prospectively enabling exploration and adjustment of patient factors associated with outcomes. Data from provinces which have not yet had outcomes published were included in analyses.

Non HIV-related co-morbidities and detailed HIV-related diagnoses are not included in the routine monitoring data collected from ART patients in South Africa, due to the increased difficulty and expense that capturing this information on a large scale would necessitate. These data elements could therefore not be included in analyses in this study. Patients with co-morbidities may be more likely to initiate ART at hospital level and also to have poorer outcomes, therefore these are potential confounders which may negatively affect overall outcome estimates at hospital level compared to PHC sites. Differences in outcomes between facilities may therefore be partially attributable to these confounders, the magnitude of which is indeterminate in this study, although it is unlikely to be large as a relatively lesser proportion of patients have serious non HIV-related morbidity or HIV-related morbidity not reflected in baseline immunological status or WHO clinical staging. Other studies comparing outcomes between health system levels have also not included these data elements in analyses [12], [13]. Research has yet to be conducted in low-income countries to determine the degree to which outcome differences between facility levels are attributable to facility characteristics or patient clinical co-morbidity at hospital levels, and would require prospective studies that capture data beyond what is collected on a routine programmatic basis.

This study is a retrospective analysis using routine data with its inherent limitations, it is likely however to be indicative of the situation at an operational level. Viral load results were available for approximately 60% of eligible patients which may bias VS estimates; however availability was three-fold higher than from a number of other sub-Saharan ART programs [30]. Over 30% of patients LTFU from ART programs have been found to have died in studies linking the vital status of patients LTFU with national death registries [31], [32]. Due to the large cohort size in this study we did not link patients LTFU with death registries, and were therefore not able to determine the proportion of unascertained deaths amongst those LTFU or to calculate corrected mortality figures. It is possible that misclassification of patients as LTFU instead of transferred-out was differentially increased at regional hospitals compared to other facilities, as regional hospitals had the highest rates of transfer-out and the highest patient loads predisposing to data capture error; this may falsely elevate LTFU at regional hospitals although the magnitude is unlikely to be large. As the sites included were not a random sample of all public ART facilities in each province, results may not necessarily be well-generalisable to the whole of each province. The sample size in Mpumalanga was small in relation to the samples from other provinces, therefore outcomes from this province should be viewed cautiously.

Household socio-economic data is not collected as part of routine monitoring of ART patients in South Africa, and there is little reliable data comparing socioeconomic indices between patients accessing care at different levels of the health system in South Africa. Socioeconomic data could therefore not be included in analyses when comparing outcomes between health system levels; previous studies investigating this question similarly did not include this type of data [12], [13]. There is however no reason to suggest that patients attending hospitals have significantly worse socioeconomic indices than patients attending PHC clinics, therefore this limitation is not expected to be a confounder of the results. Indeed, patients at PHC clinics are more likely to have worse socioeconomic indices, as PHC clinics have more rural patients than hospitals (which are mostly urban-based), and poverty is worse in rural than in urban areas [33].

In conclusion, these results suggest that ART program management at the primary level of the healthcare system in a developing country setting is equally or more effective than in a hospital-based setting. Further operational research should be conducted on the implementation of nurse-led services and potentially devolving aspects of ART care to the community. There remains a need for innovative solutions to programme expansion to increase enrolment for the growing number of patients needing treatment, manage stable patients, reduce programme costs, as well as to increase access to more effective treatments and patient monitoring systems such as viral resistance testing. The ongoing challenges faced in the struggle to scale up high-quality ART services are formidable, but addressing these effectively has the potential to considerably improve HIV/AIDS related morbidity and mortality in South Africa.
